# Insight into Elongation Stages of Peptidoglycan Processing in Bacterial Cytoplasmic Membranes

**DOI:** 10.1038/s41598-018-36075-y

**Published:** 2018-12-07

**Authors:** Seonghoon Kim, Marcos M. Pires, Wonpil Im

**Affiliations:** 1 Departments of Biological Sciences and Bioengineering, Lehigh University, 111 Research Drive, Bethlehem, PA 18015 USA; 2 Department of Chemistry, Lehigh University, 111 Research Drive, Bethlehem, PA 18015 USA

## Abstract

Peptidoglycan (PG) biosynthesis and assembly are needed for bacterial cell wall formation. Lipid II is the precursor in the PG biosynthetic pathway and carries a nascent PG unit that is processed by glycosyltransferases. Despite its immense therapeutic value as a target of several classes of antibiotics, the conformational ensemble of lipid II in bacterial membranes and its interactions with membrane-anchored enzymes remain elusive. In this work, lipid II and its elongated forms (lipid VI and lipid XII) were modeled and simulated in bilayers of POPE (palmitoyl-oleoyl-phosphatidyl-ethanolamine) and POPG (palmitoyl-oleoyl-phosphatidyl-glycerol) that mimic the prototypical composition of Gram-negative cytoplasmic membranes. In addition, penicillin-binding protein 1b (PBP1b) from *Escherichia coli* was modeled and simulated in the presence of a nascent PG to investigate their interactions. Trajectory analysis reveals that as the glycan chain grows, the non-reducing end of the nascent PG displays much greater fluctuation along the membrane normal and minimally interacts with the membrane surface. In addition, dihedral angles within the pyrophosphate moiety are determined by the length of the PG moiety and its surrounding environment. When a nascent PG is bound to PBP1b, the stem peptide remains in close contact with PBP1b by structural rearrangement of the glycan chain. Most importantly, the number of nascent PG units required to reach the transpeptidase domain are determined to be 7 or 8. Our findings complement experimental results to further understand how the structure of nascent PG can dictate the assembly of the PG scaffold.

## Introduction

Bacterial cell wall biogenesis is critically important to the viability of bacteria and underpin bacterial pathogenesis and human microbiome interactions^1^. Considering that peptidoglycan (PG) is the major component of most bacteria, the cellular machinery that processes building blocks related to the cell wall is tightly linked to PG structural integrity and cellular maintenance^2^. Moreover, molecules that disrupt PG assembly are some of the most efficacious antibiotics discovered to date^3,4^. Gaining deeper understanding into PG biosynthesis may reveal potential targets for drug discovery and development.

Lipid II is the lipid-bound precursor of PG, a primary component of bacterial cell walls. PG consists of cross-linked layers that contribute to cell shape and cell wall strength^5–7^. Lipid II consists of β-(1→4)-*N*-acetylglucosamine (GlcNAc)-*N*-acetylmuramic acid (MurNAc) disaccharide unit linked to a pentapeptide stem peptide via MurNAc. The disaccharide unit is connected to an undecaprenyl tail by pyrophosphate and the tail anchors lipid II onto the membrane. While the PG pentapeptide sequence can vary depending on the bacterial species, the canonical sequence for the majority of Gram-negative bacteria is l-Ala-γ-d-Glu-*m*-DAP-d-Ala-d-Ala, where *m*-DAP is *meso*-diaminopimelic acid^2^.

Lipid II molecules are processed by a number of membrane-anchored enzymes^4,8–10^. Among them, penicillin-binding protein 1b (PBP1b) is a member of the bifunctional class A that carries out both glycosyltransferase (GTase) and transpeptidase (TPase) activities^11^. GTase domains processes lipid II by polymerizing the glycan chains, whereas TPase domains perform covalent cross-links between neighboring stem peptides^12^. Given the importance of GTase activity for PG biosynthesis, it is important to understand how glycan chain elongation can control substrate access and priming. Towards these goals, the crystal structure of GTase from *Staphylococcus aureus* was previously solved with the antibiotic moenomycin to gain structural insight into the mechanism of action of moenomycin^13^. Moreover, the full-length *Escherichia coli* (*E. coli*) PBP1b structure containing the transmembrane helix subdomain was recently published^14^. Based on these works, we have herein set out to model nascent PG in complex with PBP1b with the ultimate goal of gaining structural insight into PBP-PG interactions.

**Figure 1:**
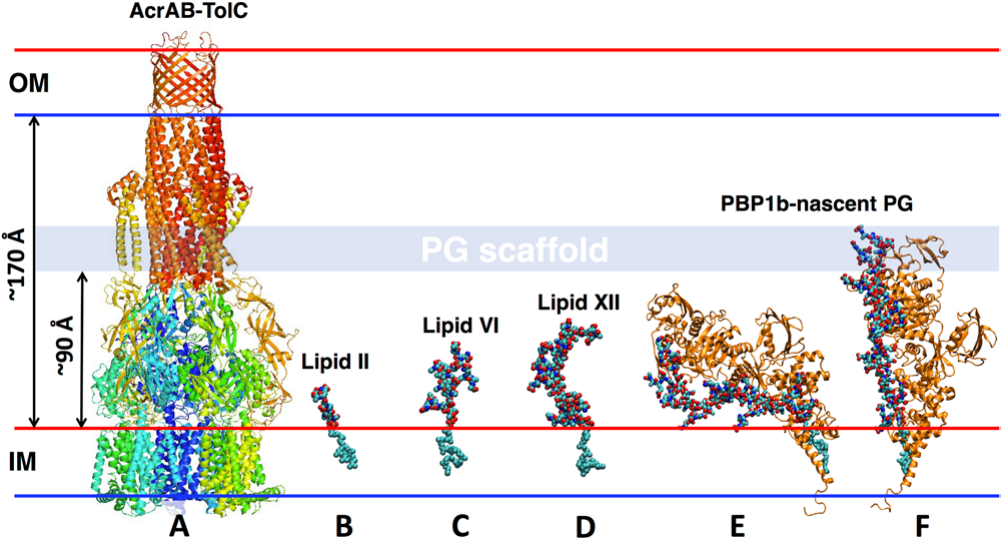
Schematic representation of the *E. coli* cell envelope with the structures of (**A**) AcrAB-TolC, (**B**) lipid II, (**C**) lipid VI, (**D**) lipid XII, and (**E**) the most tilted and (**F**) the least tilted PBP1b-lipid^XX^ complex. The AcrAB-TolC structure is used to approximately measure the vertical distance between the phosphate of the upper leaflet of the inner membrane (IM) to the phosphate of the lower leaflet of the outer membrane (OM). The periplasmic length (~170 Å) is measured by the length of AcrAB-TolC^39^, and the distance between the PG and the inner membrane is ~90 Å^40^.

**Figure 2:**
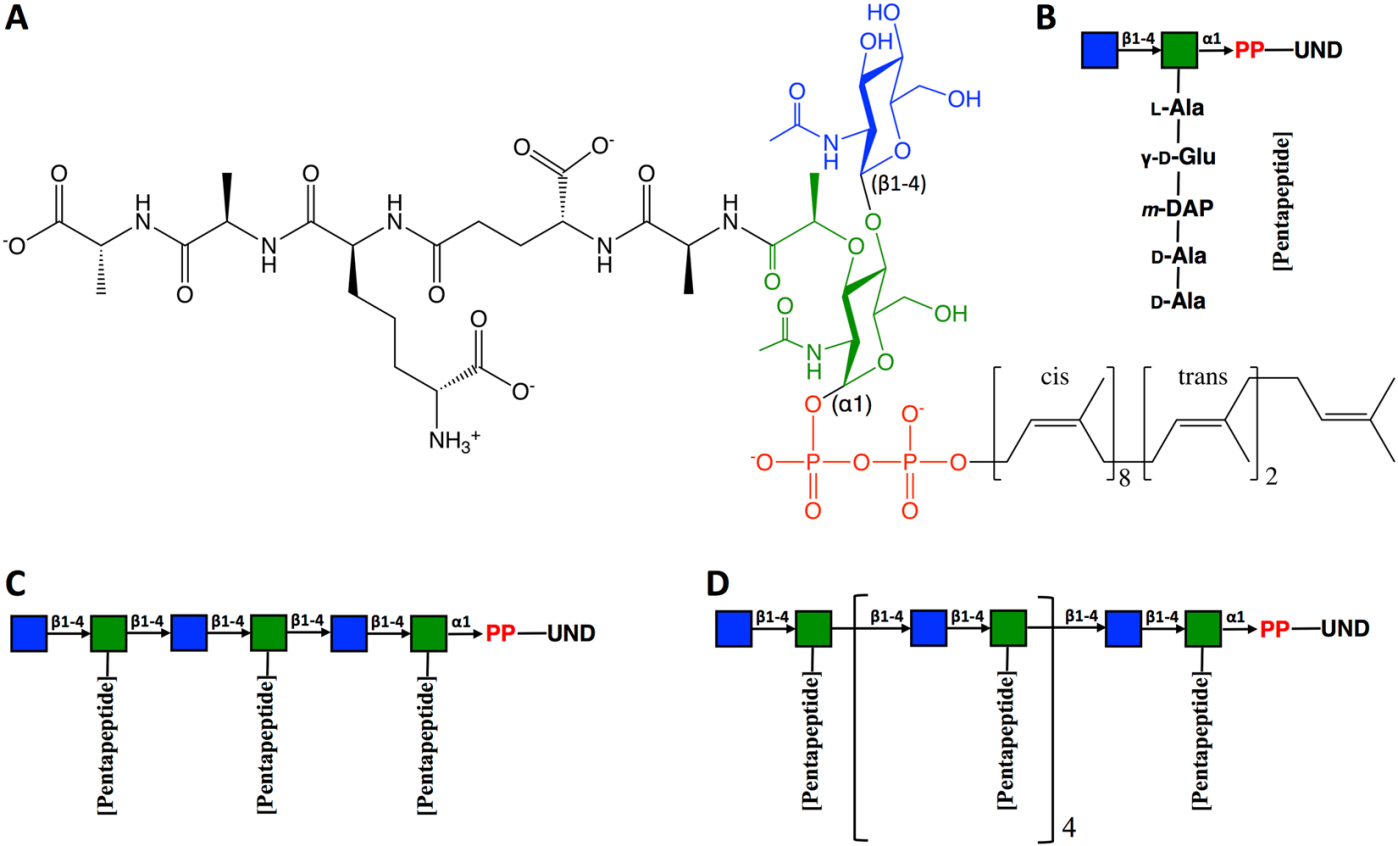
(**A**) Chemical structure of lipid II composed of MurNAc (green), GlcNAc (blue), pyrophosphate (red), undecaprenyl tail (black), and pentapeptide (black). Schematic representations of (**B**) lipid II, (**C**) lipid VI, and (**D**) lipid XII; pyrophosphate and undecaprenyl moieties are abbreviated as PP and UND, respectively.

Given the importance of lipid-bound PG precursors in PG biosynthesis and drug-discovery^4,15^, there are unanswered fundamental questions about how PG elongation affects lipid II processing by PBPs. Elegant experimental studies have described the elongation of nascent PG building blocks into extended lipid-bound precursors by PBPs^16–19^. It was established that during glycan elongation by GTase, glycan chains are stitched together unidirectionally before being loaded onto the existing PG scaffold. Yet, these studies did not provide atomistic details about the dynamics of lipid-bound PG precursors in a membrane environment and within PBPs. How do elongated lipid-anchored PG intermediates behave within bacterial membranes? How does the size of the nascent PG control association with PBPs? Most critically, how many elongation steps are needed for the nascent PG to reach the TPase domain? Towards answering some aspects of these fundamental questions, we first modeled and simulated Gram-negative lipid-anchored PG precursors such as lipid II, lipid VI, and lipid XII in membranes that are representative of Gram-negative bacteria (Figs 1 and 2). Next, *E. coli* PBP1b was modeled and simulated in the presence of nascent PG (PBP1b-lipid^XX^ complex) to gain atomistic insight into the dynamics of nascent PG in association with PBP1b (Fig. 1).

## Results and Discussion

### Isolated lipid II, lipid VI, and lipid XII in a Gram-negative Bacterial Cytoplasmic Membrane

**Figure 3:**
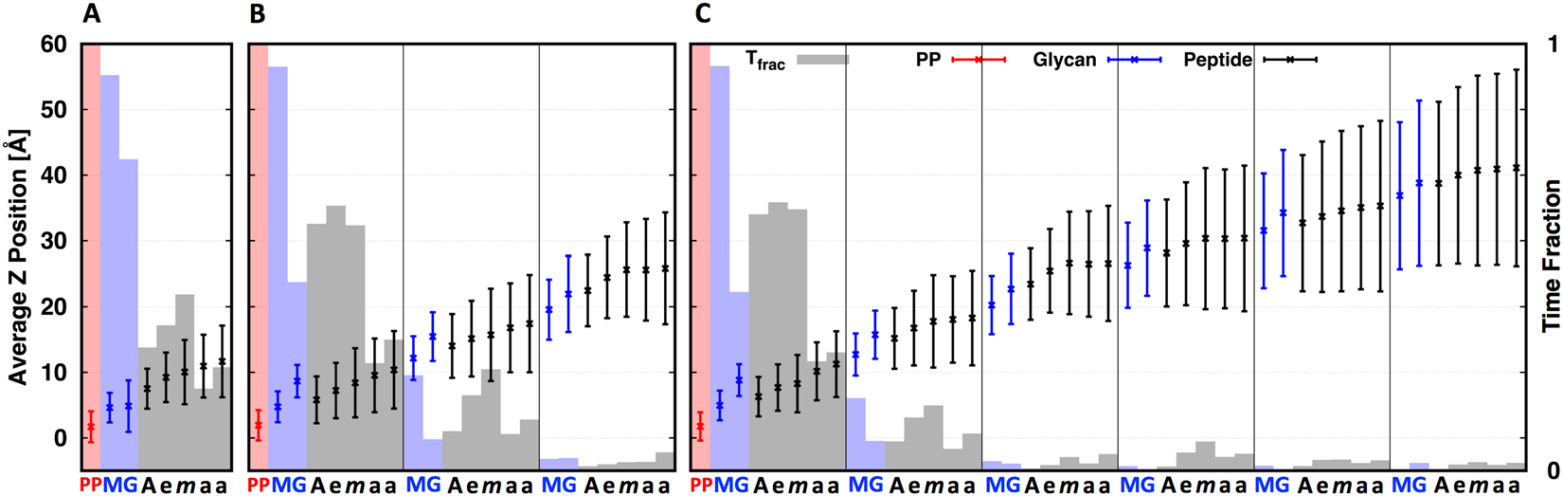
For (**A**) lipid II, (**B**) lipid VI, and (**C**) lipid XII systems, the average Z-distances (*dot*) from the lipid phosphate of the upper leaflet of a Gram-negative cytoplasmic membrane with the standard deviations (*error bar*; *n* = 4167 for the lipid II system and *n* = 8335 for the other systems), and the interaction fraction time (*box*) of the pyrophosphate (PP, *red*), glycan chain (MG, *blue*), and pentapeptide stem (black): l-Ala (A), γ-d-Glu (e), *m*-DAP (*m*), and d-Ala (a).

To characterize the conformational dynamics of isolated lipid II, VI, and XII (Fig. 2B–D) embedded in a Gram-negative cytoplasmic membrane composition (POPE:POPG = 3:1), we performed 1-μs simulation for each system (Fig. S1; Table S1; see Methods for details). We calculated the average Z-position of lipid II/VI/XII components relative to the phosphate groups of the membrane, as well as the fraction of interaction time between each residue and the membrane surface. Figure 3 shows the average Z-position (*dot*) for lipid II, VI, and XII with standard deviations over the entire trajectory. The fraction of interaction time (Fig. 3, *shaded box*) was defined as the fraction of the simulation time that each residue interacts with membrane head groups. As the length of the nascent PG increases, the non-reducing (distal) end of the glycan strand samples a larger physical space (particularly along the membrane normal). In turn, such sampling facilitates the glycan moiety to interact with lipid head groups even in lipid XII, albeit the fraction time is relatively short. During lipid II polymerization, transglycosylation occurs on the outer leaflet of the cytoplasmic membrane^18^. As shown in the following section, the GTase domain needs to bind two nascent PGs to elongate the lipid-anchored PG precursor: one in the donor site and the other in the acceptor site^20^. Thus, the non-reducing end of acceptor strand needs to access the reducing end of donor strand for the completion of the transglycosylation step. Our simulation results show that for the elongated forms of lipid II (e.g., lipid VI and XII), the non-reducing end has limited time to access to the membrane surface, which may disfavor its ability to act as the intermolecular nucleophile onto the reducing end of a nascent PG.

**Figure 4:**
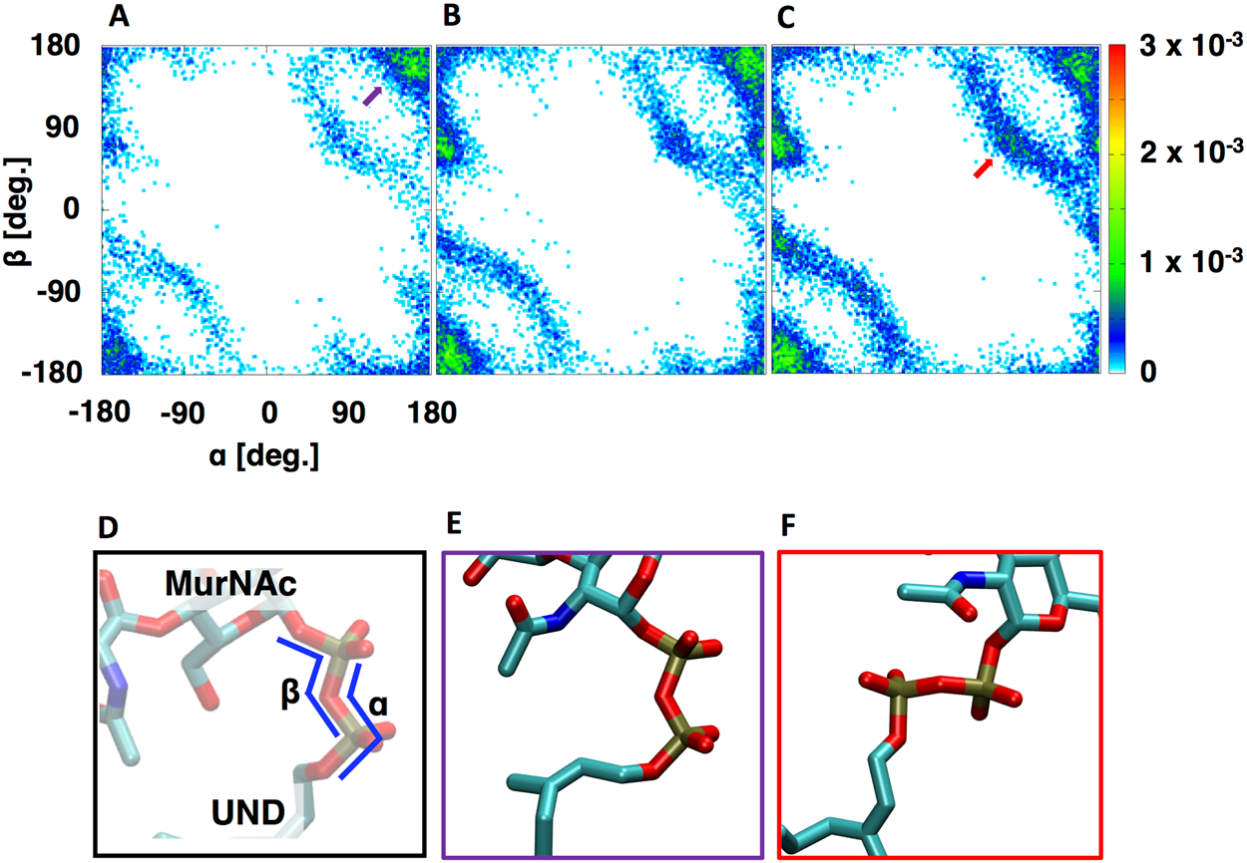
(**A**) Two-dimensional pyrophosphate dihedral angle plots of (**A**) lipid II, (**B**) lipid VI, and (**C**) lipid XII. (**D**) The two dihedral angles of pyrophosphate are defined as α (O11^UND^-P1-O12-P2) and β (P1-O12-P2-O22^MurNAc^), and (**E**) a *syn*-configuration (*purple arrow* in *A*) and (**F**) an *anti*-configuration (*red arrow* in *C*) of the pyrophosphate moiety.

Prior studies have suggested that the pyrophosphate moiety plays an important role in the binding and stabilization of lipid II within positively charged cages of various proteins that process lipid II molecules^21,22^. Likewise, several residues from GTase domains of PBPs have been observed to coordinate the pyrophosphate moiety of the lipid II-mimetic donor chain^20^. In our simulation, lipid II, VI, and XII show distinct patterns of pyrophosphate dihedral angles (Fig. 4A–C; see Fig. S2 for the characterized Newman projection of each hot spot). The distribution of dihedral angles of lipid II pyrophosphate is restricted in range (Fig. 4A). Moreover, the PG moieties such as glycan and peptide (head) and the undecaprenyl moiety (tail) of lipid II are preferably found to be on the same side (*syn-*configuration) (Figs 4E; S2). In contrast, the pyrophosphate moieties in lipid VI and lipid XII become frequently distorted (Fig. 4B,C) in that the head and tail moieties are preferably placed on different sides (*anti-*configuration) (Fig. 4F). The implication of having *syn* configurations during transglycosylation is discussed in the next section. It is known that the number of saccharide units within the lipid-anchored PG precursor plays a prominent role in GTase activity^23^. The catalytic efficiency of lipid II processing is 20–40 times greater than lipid IV, and elongated nascent PGs (longer than lipid IV) are poor GTase substrates. Our results suggest that elongated nascent PGs frequently form unfavorable pyrophosphate angles that cause *anti-*configuration relative to lipid II within the positively charged cage in GTase domains. In turn, altered angles can potentially have a significant impact on the processing efficiency of elongated nascent PGs.

### Dynamics of PBP1b associated with lipid-anchored nascent PG

**Figure 5:**
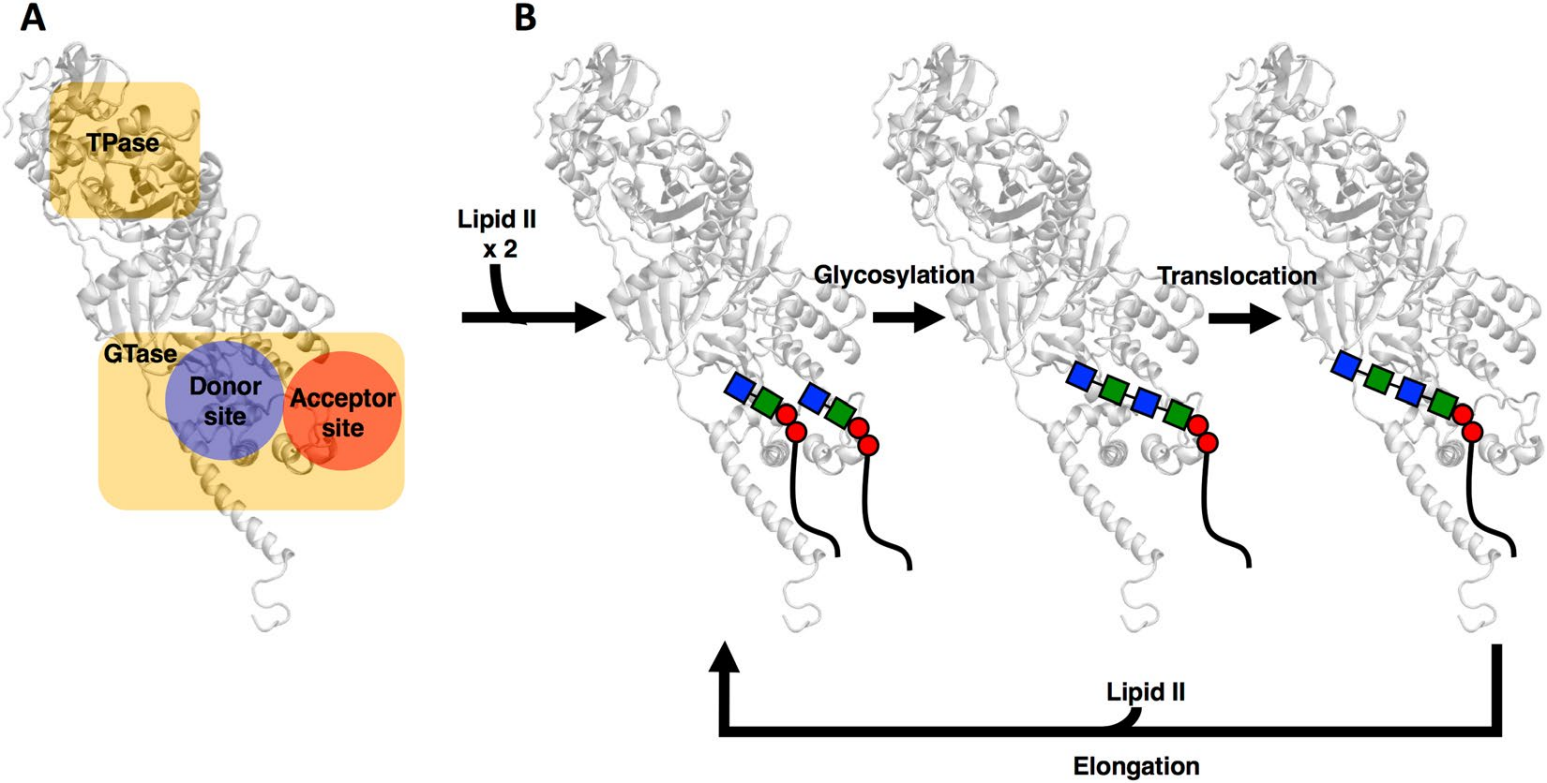
A proposed mechanism for transglycosylation of lipid II by the GTase domain in PBP1b (*white*). (**A**) Unbound PBP1b, active sites of two domains (*yellow*), and the donor (*blue*) and acceptor sites (*orange*) in the GTase domain. (**B**) PBP1b-lipid^II^-lipid^II^ ternary complex, PBP1b with turn-over product after glycosylation, and a nascent PG in the donor site after translocation.

**Figure 6:**
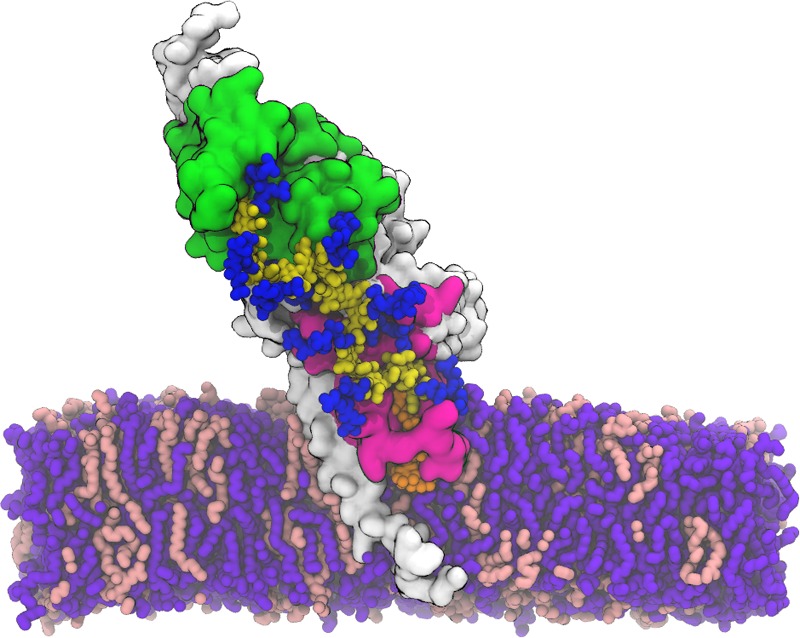
A representative snapshot of PBP1b-lipid^XX^ complex embedded in a Gram-negative cytoplasmic membrane (PE:PG = 3:1): white surface for PBP1b, magenta surface for GTase, green surface for TPase, blue sticks for pentapeptide stem, orange sticks for undecaprenyl pyrophosphate, yellow sticks for glycan chain, violet sticks for POPE, and pink sticks for POPG. Water molecules and KCl ions are not shown for clarity.

Transglycosylation of PG precursors are proposed to be carried out when two lipid-anchored PG precursors are properly placed in the donor and acceptor sites (Fig. 5A)^13^. The product of this reaction is the formation of an extended lipid-anchored PG precursor (Fig. 5B). Next, the growing nascent PG is translocated from the acceptor site to the donor site for further transglycosylation reaction(s). In this study, a PBP1b-nascent PG complex was built to represent a final state after translocation of nascent PG, which is an appropriate model to investigate the overall function of PBP1b in the elongation stage. For this purpose, antibiotics (moenomycin and aztreonam) that were originally embedded with PBP1b in the crystal structure (PDB:5HLB) were removed, and lipid XX (20 monosaccharides) was placed in the donor site (Fig. 5A*, blue sphere*). The placement of the nascent PG was based on the position of moenomycin in the X-ray structure. The PBP1b-lipid^XX^ complex was embedded into a Gram-negative cytoplasmic membrane and the entire system was simulated for 2 μs (Fig. 6; Table S1; see Methods for details).

**Figure 7:**
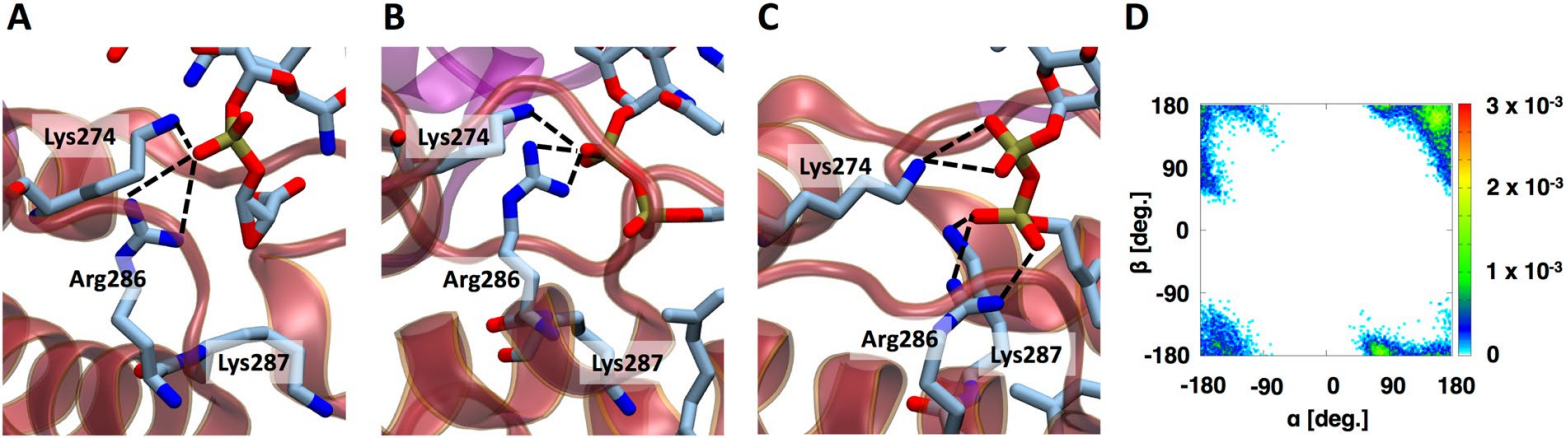
Snapshots of the pyrophosphate cage in the donor site of (**A**) PDB:5HLB with moenomycin, PBP1b-lipid^XX^ complex at the initial (**B**), and at 2 μs (**C**). (**D**) 2D pyrophosphate dihedral angle plot of lipid XX.

In the crystal structure of PBP1b, moenomycin is bound to the GTase donor site, and two positive residues in the cationic cage (Lys274 and Arg286) make salt bridges with the moenomycin phosphate moiety (Fig. 7A)^14^. These results provide critical insight into the mechanism of action of moenomycin by showing that it is actively competing against the GTase substrate. In our study, another lysine residue (Lys287) that was not initially coordinated the pyrophosphate unit (Fig. 7B) became accessible in the course of the simulation (Fig. 7C). The significance of our findings is that Lys287 is relatively more mobile than Lys274 or Arg286, suggesting that Lys287 also plays a role in substrate orientation and accessibility. Consistent with these results, it has been previously determined that mutation of Lys287 to Ala decreases GTase activity by ~40% but does not abrogate it^24^. Moreover, Lys274 and Arg286 residues have been shown to be critical for enzymatic activity as their mutation to Ala leads to the loss of GTase activity^24^. The three positive residues together form a positive cage in the donor site, which holds the pyrophosphate dihedral angles preferably in *syn* configurations (Fig. 4E) during the simulation (Fig. 7D). More specifically, a population of *syn* configurations is about 74% in the PBP1b-lipid^XX^ complex (Fig. S3). These findings imply that the cationic cage in GTase is suitable for preferably maintaining the pyrophosphate moiety as *syn*-configurations, which is speculated to be appropriate configuration for the transglycosylation reaction and the favorable configuration for the accessibility of lipid-anchored PG-precursor.

**Figure 8:**
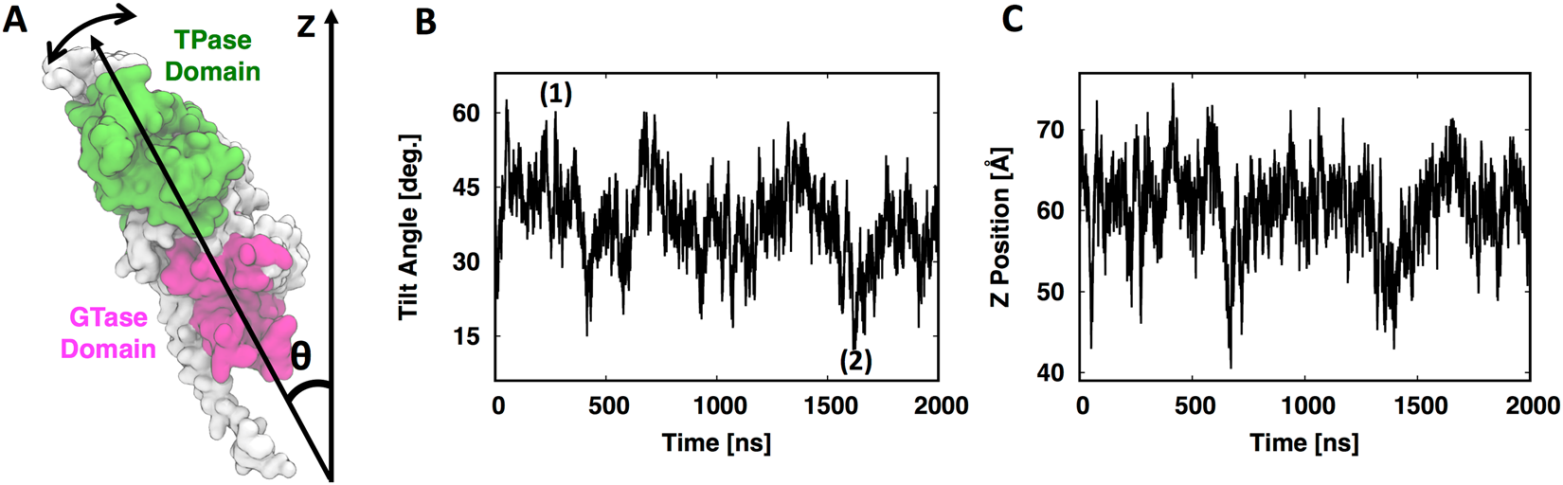
(**A**) Tilt angle with respect to the Z axis measured by the vector connecting the centers of mass of the GTase and TPase domains. (**B**) Time-series of tilt angle of PBP1b; 1 and 2 correspond to Fig. 1E and F. (**C**) Time-series of Z-position of the TPase domain’s center of mass from the upper phosphate of the upper leaflet of a Gram-negative cytoplasmic membrane.

Next, we set out to explore the conformational dynamics and interactions of the PBP1b-lipid^XX^ complex in a Gram-negative cytoplasmic membrane. During the simulation, a tilting motion of the entire protein axis is observed (Fig. 8A); in other words, the tilt angle of PBP1b with respect to the Z-axis (the membrane normal) changes from 12° to 60° frequently as a function of simulation time (Fig. 8B1,2). This tilting motion results in the effective samples of the TPase domain across 30 Å along the Z-axis (Fig. 8C). Such a large spatial sampling potentially allows for the TPase domain of PBP to interact with the existing PG for the transpeptidation reaction onto the growing lipid-anchored nascent PG (Fig. 1F). The GTase site is near the base of PBP. Thus, for the nascent PG to be stitched onto the existing PG, the backbones of the sugar units have to be connected. The tilting motion may provide the accessibility necessary for the large existing PG to reach or dock onto the base of PBP to reach the GTase active site.

**Figure 9:**
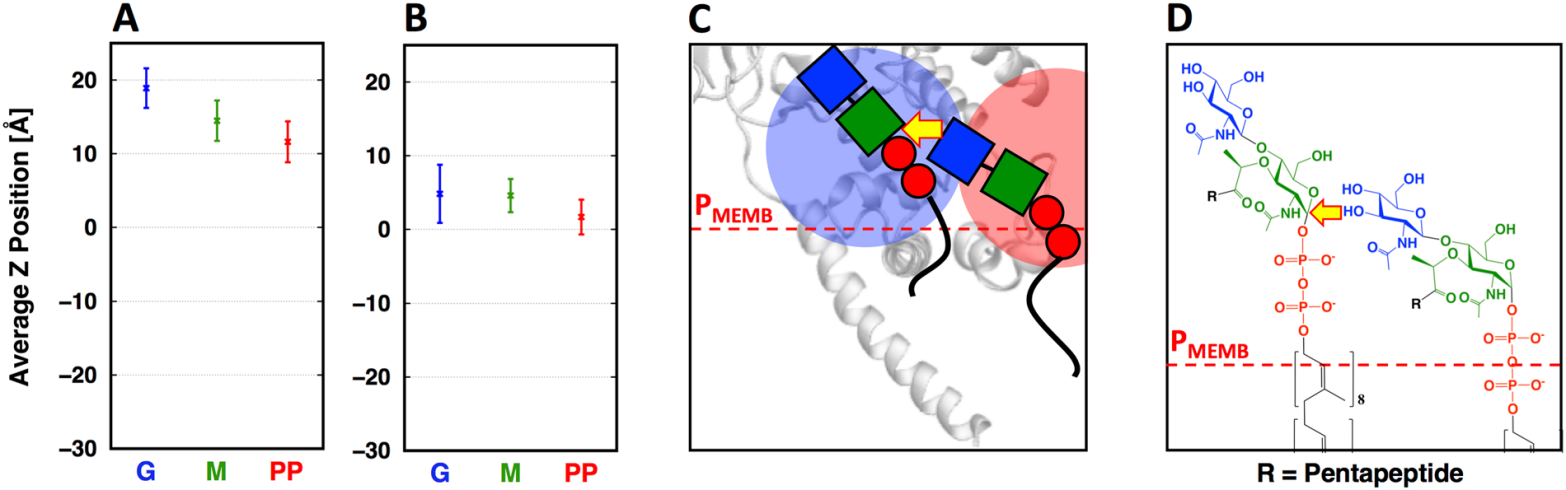
Average Z-distances with standard deviations (*error bar*) of pyrophosphate (red), MurNAc (green), and GlcNAc (blue) of (**A**) the bound nascent PG (in the PBP1b-lipid^XX^ complex) and (**B**) the isolated (unbound) lipid II from the lipid phosphate Z-position. (**C**) A schematic representation of a nascent PG in the donor site (blue circle), and lipid II in the acceptor site (red circle) of PBP1b with the Z-position of phosphate head groups (red dotted line). (**D**) A proposed mechanism for lipid II polymerization by PBP1b.

While the GTase domain has two separate substrate binding sites, both donor and acceptor sites are connected continuously through a glycan binding groove for the transglycosylation enzymatic activity. Thus, two lipid II molecules need to properly dock into the donor and acceptor sites. In our simulation, the Z-position of bound nascent PG in the donor site (Fig. 9A,C, *blue circle*) is raised approximately 10 Å relative to isolated (unbound) lipid II (Fig. 9B) with the assumption of a lipid II molecule at the acceptor site (Fig. 9C*, red circle*). We speculate that the glycan binding groove coordinates the Z-positioning of two substrates, and bind to all glycan strands collinearly. Most importantly, our findings suggest that the association of PBP1b with lipid II serves to properly align the nucleophilic attack of the non-reducing end at the acceptor on the reducing end at the donor (Fig. 9D).

**Figure 10:**
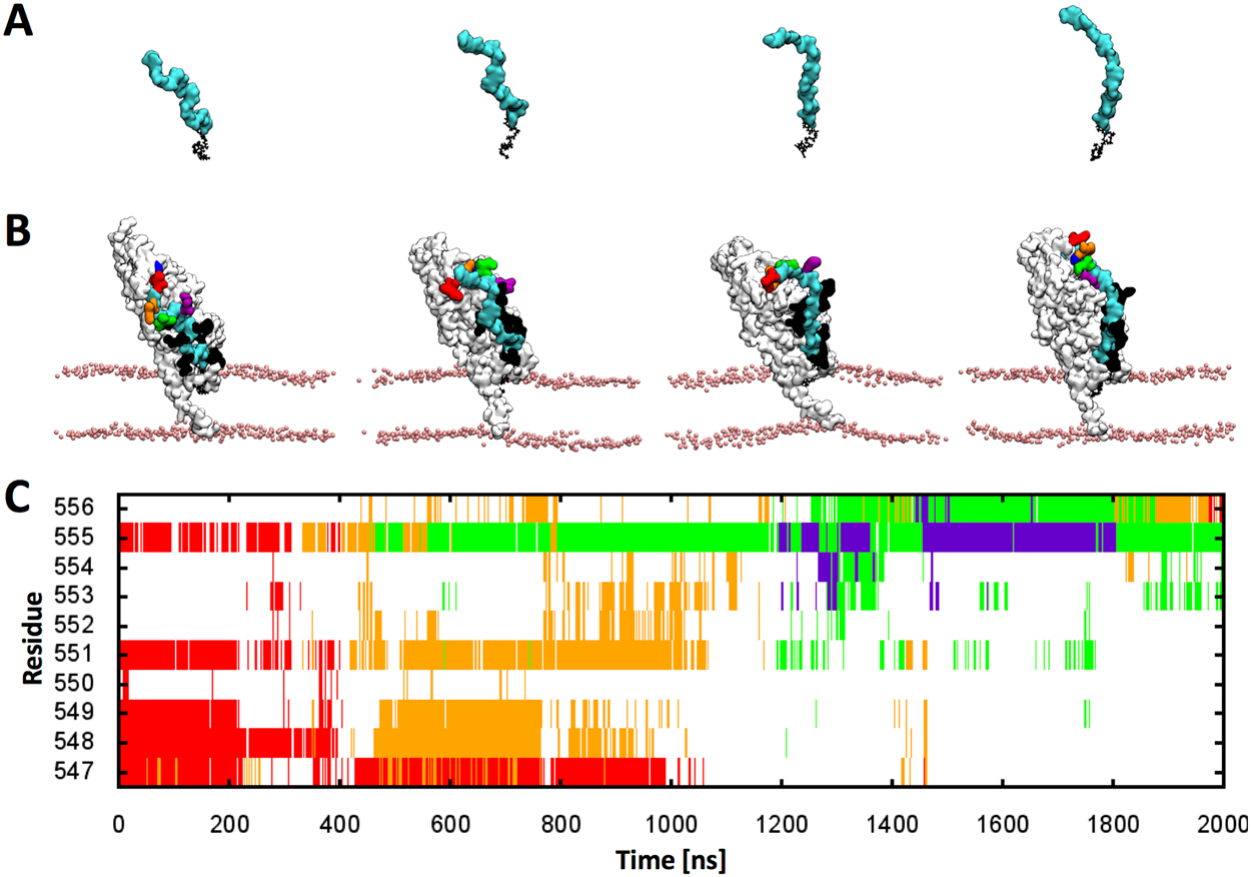
A series of (**A**) glycan chain snapshots and (**B**) snapshots of PBP1b-lipid^XX^ complex as the simulation progresses (cyan for glycan chain; white for PBP1b; blue for Arg555; pink for phosphate in the membrane; purple, green, orange, and red for 7^th^–10^th^ pentapeptide chains). (**C**) Interaction patterns of 7^th^–10^th^ peptide stems with the TPase domain residues from 547 to 556.

At the initial stage of the transglycosylation, the length of the growing nascent PG is too short to reach the TPase domain. Therefore, several transglycosylation reactions would need to occur prior to processing by the TPase domain. In our simulation, the twisted glycan strand at the beginning of the simulation becomes more stretched out and makes extensive contact with the PBP1b surface as the simulation progresses (Fig. 10A,B), leading to a higher frequency of interactions between the pentapeptide stems and TPase site (Fig. 10C). During the simulation, the 7^th^ and 8^th^ pentapeptide stems (*green* and *purple* in Fig. 10B,C) migrated and bound to the TPase site (Fig. 10B*, blue* Arg555). Presumably electrostatic forces are driving the interaction between the negatively charged nascent PG (Fig. S4A) and the positively charged surface of PBP1b (Fig. S4B). Based on our results, it was determined that approximately 7 or 8 repetitive transglycosylation reactions are required for a distal pentapeptide stem to reach the TPase site.

## Conclusions

We have investigated the structure and dynamics of isolated lipid II, its elongated forms (lipid VI and XII), and the PBP1b-nascent PG complex in Gram-negative bacterial-mimetic membranes to understand how nascent PG structures control PG elongation and processing. Lipid II needs to be polymerized by the GTase domains of PBPs for the proper assembly of PG. The GTase domain of PBP1b is located near the membrane surface, which can impose some structural constraints on the substrate structure. More specifically, the non-reducing end of lipid II in the acceptor site must also be present near the membrane surface for a productive transglycosylation reaction with another nascent PG in the donor site.

As glycan chains of lipid-anchored PG precursors are extended, while fluctuations of the non-reducing end (particular along the membrane normal) appear to increase, the non-reducing end remains distant from the membrane surface and would likely reduce the probability that its elongated nascent PG units are used as an acceptor strand. Glycan chain length also alters the dihedral angle distributions of the pyrophosphate moiety, which could be critical during glycan transfer. During our 2-μs simulation, PBP1b-nascent PG complex shows a prominent tilting motion. As a result, the Z-position of the TPase domain is varied considerably, which can contribute to the binding of the nascent PG with the large existing PG or any of the stem peptides within the growing nascent PG. The non-reducing end of the bound nascent PG is found to interact with the TPase domain for the duration of the simulation, and 7 or 8 elongation steps are needed for a pentapeptide stem to reach the TPase site in PBP1b.

## Methods

A lipid II molecule was assembled (Fig. 2A,B) to represent the prototypical Gram-negative PG precursor with *m*-DAP in the 3^rd^ residue^25^. In addition, two elongated forms (lipid VI and lipid XII) were modeled (Fig. 2C,D). All saccharides in lipid II/VI/XII were linked with β-(1→4) glycosidic bond, and our naming scheme is based on the number of monosaccharide units^26^. POPE/POPG mixed bilayer were built to mimic the prototypical Gram-negative cytoplasmic membrane composition (a ratio of PE:PG = 3:1)^27^, and each lipid II/VI/XII was embedded in the upper leaflet of the bilayer (Fig. S1; Table S1).

A previously published X-ray structure of PBP1b from *E. coli* (PDB:5HLB) was used to model a lipid anchored nascent PG with PBP1b^14^. For our modeling, the PBP1b-nascent PG complex was built to represent a final state after translocation of nascent PG. Antibiotics (moenomycin and aztreonam) in the X-ray structure were removed, and lipid XX was placed in the donor site (Fig. 5A*, blue sphere*) as a nascent PG based on the position of moenomycin in the X-ray structure. The PBP1b-lipid^XX^ complex was embedded into a Gram-negative cytoplasmic membrane (Fig. 6; Table S1).

CHARMM-GUI *Membrane Builder* protocols were used to build all systems and to prepare the necessary input files for equilibration^28–30^. In all simulations performed in this study, the CHARMM36 force field was used for proteins^31^, carbohydrates^32,33^, and lipids^34^. Also, SHAKE algorithm^35^ was applied to all bonds containing hydrogen atoms, and temperature and pressure were held at 310.15 K and 1 bar, respectively. For each system, the well-validated *Membrane Builder*’s six-step equilibrations were initially performed for 375 ps by gradually reducing dihedral (saccharide and undecaprenoid) and planar (phospholipid and water) harmonic restraints using CHARMM^36^. During the gradual equilibration steps, Langevin dynamics was used for 75 ps with NVT (constant particle number, volume, and temperature) and it was subsequently changed to the following 300 ps with NPT (constant particle number, pressure, and temperature) maintained by Hoover thermostat and Langevin piston. The additional 30-ns NPT equilibrations were performed using NAMD^37^ to further equilibrate the systems before 1-μs (for lipid II system) and 2-μs (for the other systems) ANTON^38^ simulations for the data presented in this paper. For Anton simulations, the *Multigrator* integrator was used for NPT ensemble, which controls the pressure and temperature using the Nosé-Hoover method. A 2-fs time step was used, and simulation frames were saved every 240 ps.

## Electronic supplementary material

Supplementary Information
