# Supplementary material for: Insight into Elongation Stages of Peptidoglycan Processing in Bacterial Cytoplasmic Membranes
Source: Sci Rep. 2018 Dec 7;8:17704. doi: 10.1038/s41598-018-36075-y (PMC6286386; doi:10.1038/s41598-018-36075-y)

Supplementary Information

Insight into Elongation Stages of Peptidoglycan Processing in Bacterial Cytoplasmic Membranes

Seonghoon Kim[1], Marcos M. Pires[2], and Wonpil Im[1]*

[1]Departments of Biological Sciences and Bioengineering; [2]Department of Chemistry, Lehigh University, 111 Research Drive, Bethlehem, PA 18015, USA

Table S1. System information.

	Systems	Lipid composition (ratio)	Nascent PG # mono-saccharide	Nascent PG # penta-peptide	Initial system size (Å)	# Atom	# Ions (K/Cl)	# Water
Gram-negative	Lipid II	POPE/POPG (3:1)	2	1	90×90×84	70,439	102/32	12,296
	Lipid VI		6	3	90×90×106	87,084	122/48	17,745
	Lipid XII		12	6	90×90×124	100,497	140/60	22,075
	PBP1b-lipid^{XX}		20	10	180×180×178	581,711	566/288	144,361

Figure S1. Representative snapshots of (A) lipid II, (B) lipid VI, and (C) lipid XII embedded in a Gram-negative cytoplasmic membrane (POPE:POPG = 3:1) with blue sticks for pentapeptide stem, orange sticks for undecaprenyl pyrophosphate, cyan sticks for glycan chain, violet sticks for POPE, and pink sticks for POPG. Water molecules and KCl ions are not shown for clarity.

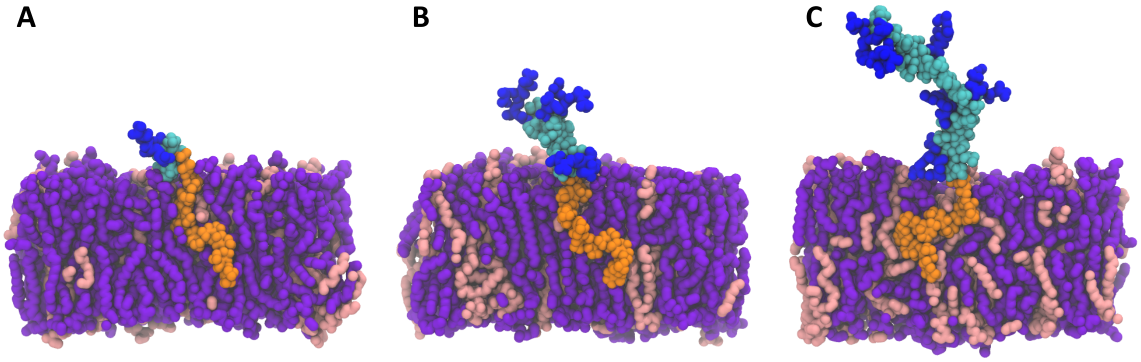

Figure S2. (A) Two-dimensional pyrophosphate dihedral angle plot of PG precursor with spots (1~5) that correspond 1 to 5 Newman projections; α ($O11^{UND}$–P1-O12-P2) and β (P1-O12-P2-$O22^{MurNAc}$).

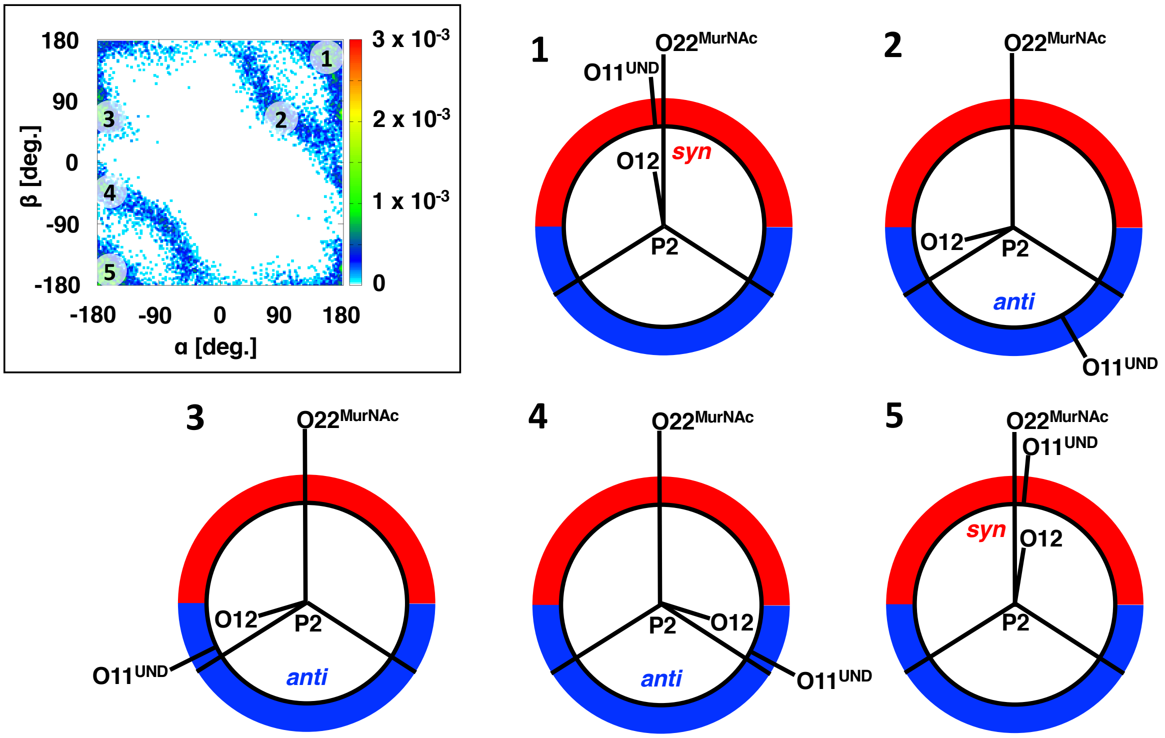

Figure S3. (A) The configuration of the nascent PG is defined by measuring the dihedral angle of the pyrophosphate moiety (O22^{MurNAc}-P2-P1-O11^{UND}). (B) The corresponding Newman projection; A torsion angle between -90° and 90° is chosen for *syn*-configurations, otherwise *anti*-configurations. The dihedral angle distributions of (C) PBP1b-lipid^{XX} complex, (D) lipid II, (E) lipid VI, and (F) lipid XII.

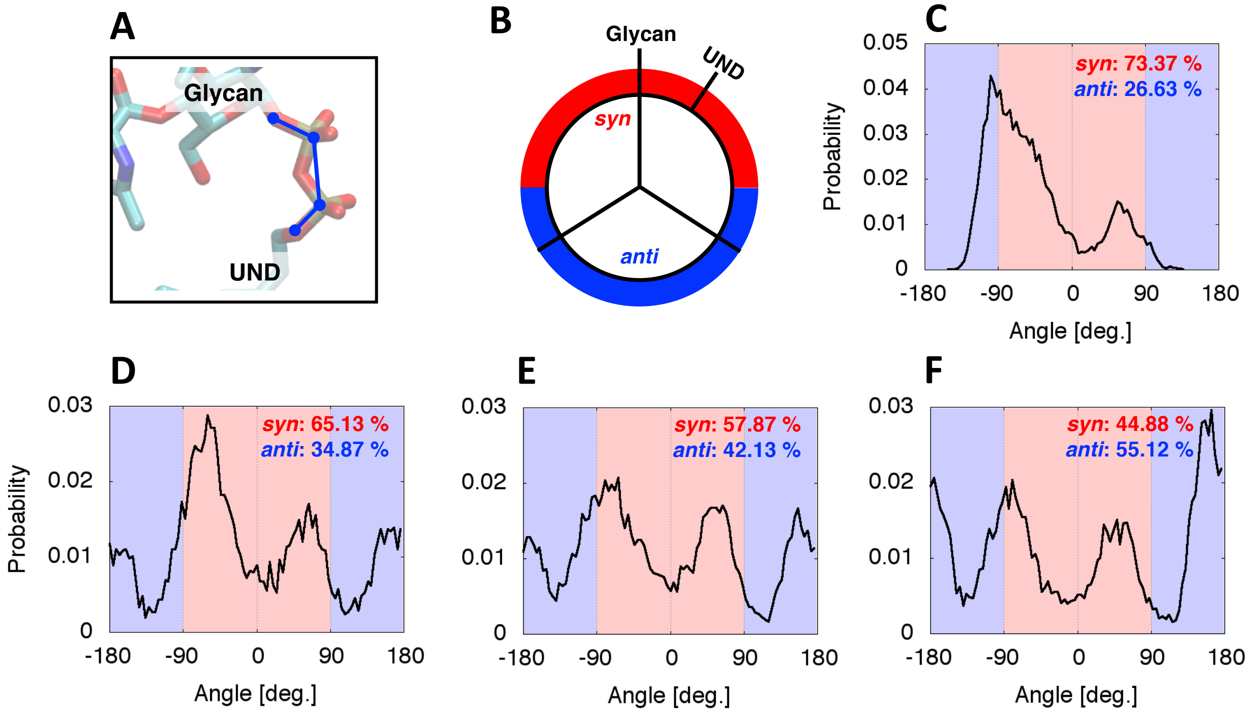

Figure S4. Solvent-accessible surface representations of (A) Gram-negative lipid XX, and (B) PBP1b with the electrostatic potential [+1 kcal/(mol·*e*) in blue to -1 kcal/(mol·*e*) in red].

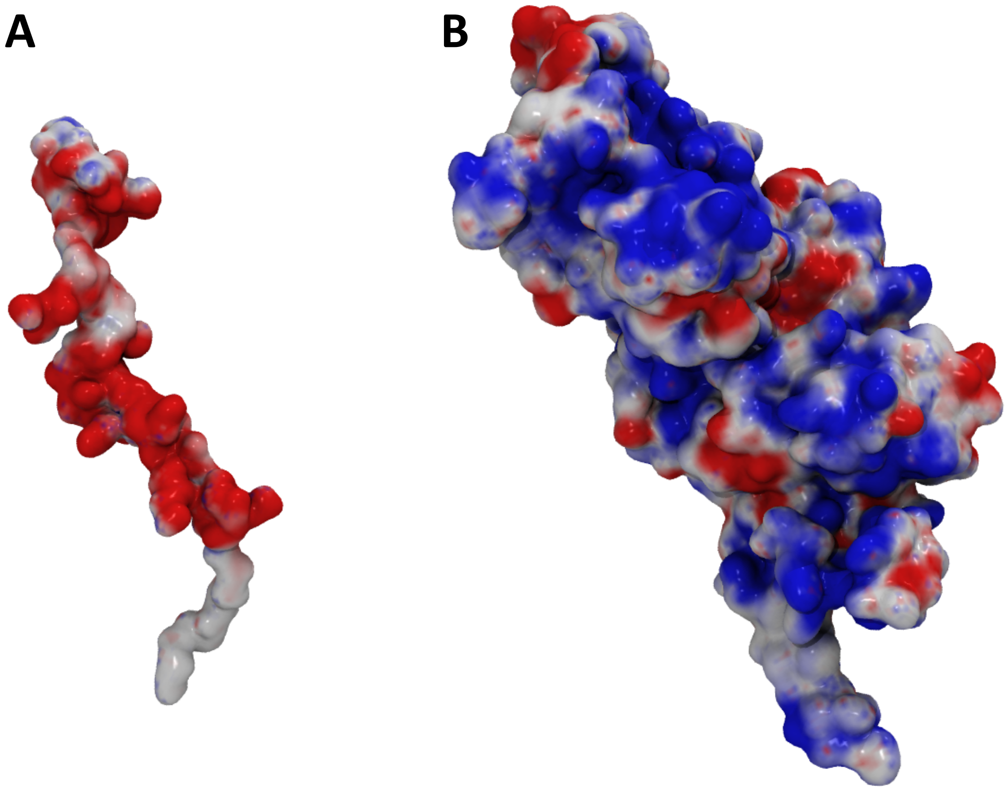

Supplement: Supplementary file 1 — Supplementary Information [file 41598_2018_36075_MOESM1_ESM.pdf]
